# Common Digital Extensor Tendon Injury in Three Sporting Dogs

**DOI:** 10.3390/ani12192619

**Published:** 2022-09-29

**Authors:** Alessio Franini, Maria Grazia Entani

**Affiliations:** Clinica Veterinaria Sporty Dog, 25125 Brescia, Italy

**Keywords:** sporting dogs, common digital extensor tendon, sport-related injuries, veterinary sports medicine, tendon injuries, ultrasound

## Abstract

**Simple Summary:**

Injuries to sporting dogs are becoming frequent, and precise knowledge of all of the anatomical structures involved in sport-related traumas is mandatory to achieve the optimum treatment for each case. In this case report, injury to the common digital extensor tendon of digits III and IV of the forelimb is reported for the first time, highlighting analogies with a similar pathology described in human athletes, called boxer’s knuckle.

**Abstract:**

Injuries to canine athletes are becoming more and more frequent, and perfect knowledge of all injury-prone anatomic structures is mandatory to achieve correct diagnoses and treatments. In this small case series discussion, trauma-based injury to the common digital extensor tendon of digits III and IV of the forelimb is described for the first time. The anatomy as well as the clinical and ultrasonographic findings show similarities to the human spectrum of pathologies called boxer’s knuckle. The treatment options with a buddy taping technique led to a satisfactory outcome at three months from trauma, without a history of re-injury. Injuries to the extensor compartment of the forepaw should be considered in cases of forelimb lameness with dorsal metacarpophalangeal swelling in sporting patients, especially with a history of blunt force traumas.

## 1. Introduction

Injuries of the common digital extensor tendon (CDET) in sporting dogs are not described in veterinary literature, but the detection of swelling of the soft tissues at the dorsal aspect of the metacarpo-phalangeal (MCP) joint, in association with different degrees of lameness as a result of trauma occurred during sporting activities, suggests the involvement of this anatomical structure and some analogies with a pathology, described in human athletes, called “boxer’s knuckle”.

The initial preliminary reports of boxer’s knuckle in human medicine date back to 1957, when Gladden described this pathology for the first time, reporting it in four cases [1]. As the name suggests, the pathology is typical of athletes who engage in sports such as boxing or martial arts, which involve punching or breaking boards of wood (for example) with their clenched fists [2].

The common digital extensor muscle arises on the lateral epicondyle and runs distally, passing through the lateral distal sulcus of the radius. Its distal CDET divides into four distinct tendons, progressing distally after crossing the dorsal surface of the carpal joint. Each tendon, from digit II to digit V, glides on the roll-like dorsal part of the corresponding metacarpal bones and is deeply embedded in the fibrous tissue of the digits. A sesamoid bone, not present in all dogs, is embedded in the tendon at the level of the MCP joint. At the distal end of the proximal phalanx, each tendon bilaterally receives a thin extension of the tendon of insertions of the interosseous muscles that crosses obliquely from the palmar surface [3].

In people, the dorsal hood is a retinacular structure that stabilizes the digital extensor tendon at the dorsal aspect of the MCP joint, keeping the tendon in place during flexion and extension. The sagittal band is the most important part of the dorsal hood, and its function is to maintain the stability of the extensor tendon [4]. Rupture of the sagittal band with subluxation or overt dislocation of the central extensor tendon is called boxer’s knuckle and mainly involves the MCP joint of the middle finger [5]. Injuries to the extensor mechanism of the fingers can be disabling and career-ending in professional human athletes [6].

To date, no descriptions of injuries of the CDET are available in the veterinary literature and, to the best of the authors’ knowledge, this is the first case report about this topic.

In the present small case series discussion, injury of the common digital extensor tendon and its peritendinous soft tissues of three sporting dogs is described, and similarities to the human condition called boxer’s knuckle are described.

## 2. Case Details

All of the following cases were presented to the Sporty Dog Veterinary Clinic (Brescia, Italy). All of the orthopedic evaluations and the radiographic assessment were double checked by two expert veterinary orthopedics (A.F. and M.G.E.). The lameness assessment for each case was based on the previously published evaluation system and is reported in Table 1 [7].

Radiographic examinations of both manus on the dorsopalmar and mediolateral standard views were performed. All of the radiographic examinations were performed under general anesthesia conducted according to a previously published protocol for orthopedic examinations. Dexmedetomidine (5 µg/kg) and butorphanol (0, 1 mg/kg) intravenously (IV), followed by propofol (1–4 mg/kg IV to effect) were used. The owners agreed to the procedure by signing a written informed consent form [8]. All of the ultrasonographic evaluations were performed by the same operator (M.G.E.) using an 18 MHz linear tranducer probe (MyLab Sigma VET, Esaote, Italy). A complete examination of the CDET of digits III and IV was performed using both longitudinal and transverse scans. The focal zone, imaging depth, and postprocessing parameters were optimized for musculoskeletal imaging.

### 2.1. Case 1

A 17-month-old female Whippet competing in lure-coursing activity presented with an episode of lameness to the right forelimb. Symptoms had been observed for two days. The lameness occurred during a lure-coursing performance and blunt trauma on uneven terrain was reported by the owner.

#### 2.1.1. Clinical Examination

Upon clinical examination, the dog was found to be in good general health. Orthopedic examination showed no lameness at a walk, but grade two lameness was detected at a trot and gallop. Swelling of the dorsal aspect of the IV MCP joint was detected and pain at palpation of the swollen region was evident. Pain at passive forced complete flexion and extension of digit IV at the level of the MCP joint was also detected. Flexion and extension of the MCP joint were reduced. No signs of instability were detected at the palpation on the lateral nor on the medial aspect of the MCP joint and all of the interphalangeal joints of the affected digit.

#### 2.1.2. Radiographic Examination

No changes were detected at the radiographic examination.

#### 2.1.3. Ultrasonographic Examination (US)

The US exam showed swelling of the peritendinous soft tissues on the dorsal aspect of the MCP joint of digit IV, with normal echogenicity of the digital extensor tendon. Disruption of the peritendinous fibrous tissue was evident at the level of the joint with irregular echogenicity of the surrounding tissue. A clear anechoic layer was present around the tendon that appeared grossly intact. At the dynamic evaluation, with passive flexion and extension movement, the tendon was always in a physiological position. Thus, no luxation or subluxation of the tendon were suspected. The CDET and the peritendinous soft tissues of digit III were within normal limits from the ultrasonographic point of view. Based on the US findings, a suspected diagnosis of lesion of the peritendinous soft tissues on the dorsal aspect of the MCP joint was considered.

### 2.2. Case 2

A male Rhodesian Ridgeback aged six years and four months old competing in canicross activity, presented with recurrent lameness to the left forelimb. Symptoms had been observed for two months. An improvement with rest was reported by the owner and worsening with sporting activity was also reported. The first episode of lameness occurred the day after a canicross performance and the owner referred to a blunt trauma against a root found on the racetrack.

#### 2.2.1. Clinical Examination

Upon clinical examination, the dog was found to be in good general health. Orthopedic examination showed no lameness at a walk, but grade two lameness was identified during a trot and gallop. Swelling of the dorsal aspect of the IV MCP joint of the left manus was detected and mild pain at palpation of the swollen region was present. A slight swelling of the dorsal region of the MCP joint was also observed at the level of digit III and no pain was detected on palpation of this region. Pain at passive forced complete flexion and extension of digit IV at the level of the MCP joint was present, whereas no pain at the same manipulation of digit III at the level of MCP joint was observed. The range of motion of the MCP joint was reduced in the flexion and extension of digit IV, whereas it was apparently within normal limits for the MCP joint of digit III. No signs of instability were detected on the lateral nor the medial aspect of the MCP joint and of the interphalangeal joints of the involved digits.

#### 2.2.2. Radiographic Examination

No significant changes were detected at the radiographic examination.

#### 2.2.3. Ultrasonographic Examination

The US exam showed digit IV swelling of the peritendinous soft tissues on the dorsal aspect of the MCP joint, with normal echogenicity of the digital extensor tendon. Disruption of the peritendinous fibrous tissue was evident at the level of the joint, with irregular echogenicity of the surrounding tissue. At the dorsal aspect of the MCP joint of digit III, an irregularly echogenic area was present around the CDET that was intact, with normal echogenicity and no evidence of core lesions. A clear anechoic layer was present around the tendons of digits III and IV. At the dynamic evaluation, with passive flexion and extension movement, clear snapping of both tendons was not detectable. Thus, no luxation or subluxation of the tendons were suspected. The CDET and the peritendinous soft tissues of digit II were within normal limits from the ultrasonographic point of view.

Based on the US findings, a suspected diagnosis of lesion of the peritendinous structures on the dorsal aspect of digit III and IV at the level of MCP joint was considered.

### 2.3. Case 3

A male Shetland Sheepdog aged eight years and two months old involved in high-level agility competitions presented with persistent lameness to the right manus. The lameness had occurred two weeks prior, and subsequent to an agility competition. Trauma involving a tunnel approach during the performance was reported by the owner. No improvement with rest was achieved and worsening at the return-to-work was observed.

#### 2.3.1. Clinical Examination

Upon clinical examination, the dog was found to be in good general health. Orthopedic examination showed no lameness at a walk, but a grade two lameness was identified at a trot, worsening to grade three at a gallop. Clear swelling of the dorsal aspect of the MCP joint of digit IV of the right forelimb was observed, with pain at palpation of the swollen region. Pain at passive forced complete flexion and extension of digit IV was detected and a clear snapping sensation at the gliding of the tendon in complete flexion was noticed (Supplementary Material, Video S1). The range of motion of the MCP joint of digit IV was reduced in flexion and extension and no signs of instability were detected in the medial nor in the lateral direction.

#### 2.3.2. Radiographic Examination

Radiographic examination was within normal limits.

#### 2.3.3. Ultrasonographic Examination

The US exam showed digit IV swelling of the peritendinous soft tissues on the dorsal aspect of the MCP joint, with normal echogenicity of the CDET. Disruption of the peritendinous fibrous tissue was also detected at the level of the joint, with irregular echogenicity of the surrounding tissue. The CDET of digit IV was intact, without signs of core lesions. A clear anechoic layer was present around the tendons of digit IV. At the dynamic evaluation, with passive flexion and extension movement, clear snapping of the CDET of digit IV was detectable and the tendon shifted medially slightly during complete flexion of the joint. The CDET and peritendinous soft tissues of digit III were within normal limits from the ultrasonographic point of view. Based on the US findings, a subluxation of the CDET of digit IV with lesion of the peritendinous tissues was suspected.

## 3. Treatment Options and Outcomes

A conservative approach was chosen as the treatment option for all three cases. The involved digit was immobilized in extension for four weeks using a “buddy taping” technique, according to previously published literature [9]. In the first four weeks, rest and leash walk were prescribed. After that, free walks were allowed twice a day for four weeks. Sharp turns and fast running were avoided, and passive range of motion associated with proprioceptive exercises was suggested. After eight weeks, the patients were clinically evaluated by both authors. No pain on flexion and extension of the involved digits was detected, swelling of the soft tissue was absent, and no functional impairment was present. After three months, a telephonic follow-up was conducted, and all of the patients were back to the sporting activity without re-injuries.

**Figure 1:**
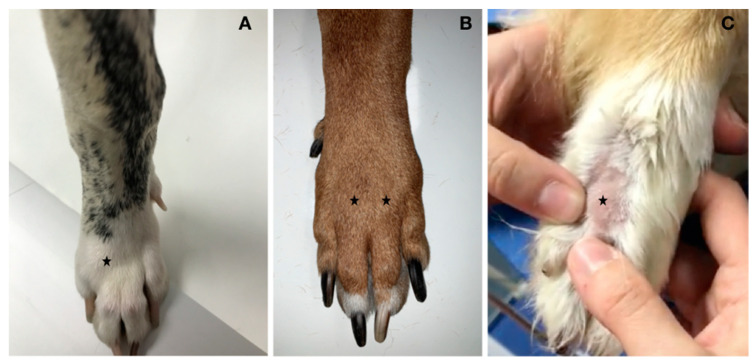
The appearance of the paws of cases 1, 2, and 3. (**A**) Case 1 showed clear swelling of the dorsal aspect of the forepaw, in correspondence with the MCP joint of digit IV (black star). (**B**) Case 2 showed mild swelling of the dorsal aspect of the forepaw, in correspondence with the MCP joint of digits III and IV (black star). (**C**) Case 3 showed mild swelling of the dorsal aspect of the forepaw, in correspondence with the MCP joint of digit IV (black star).

Figure 1 shows the appearance of the paws of the three patients.

**Figure 2:**
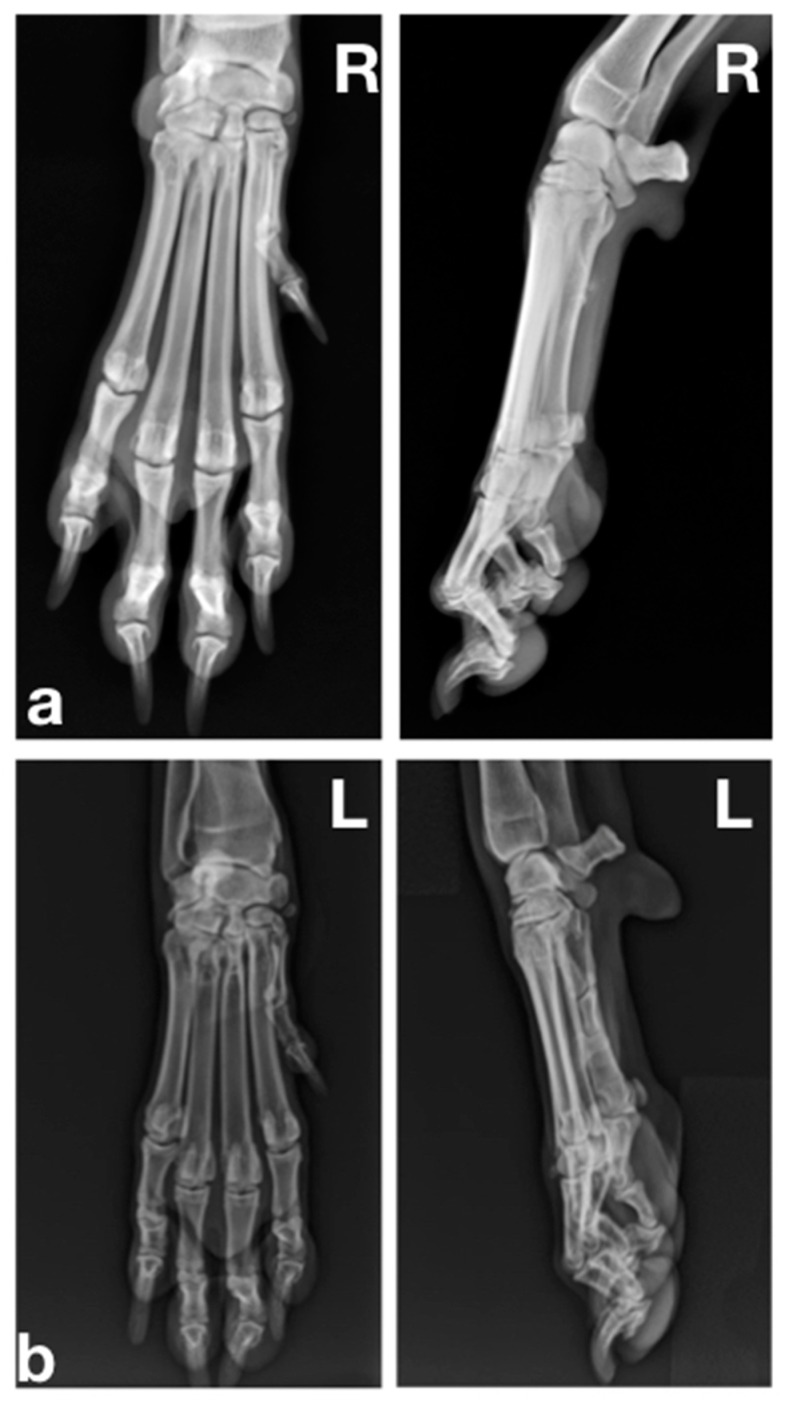

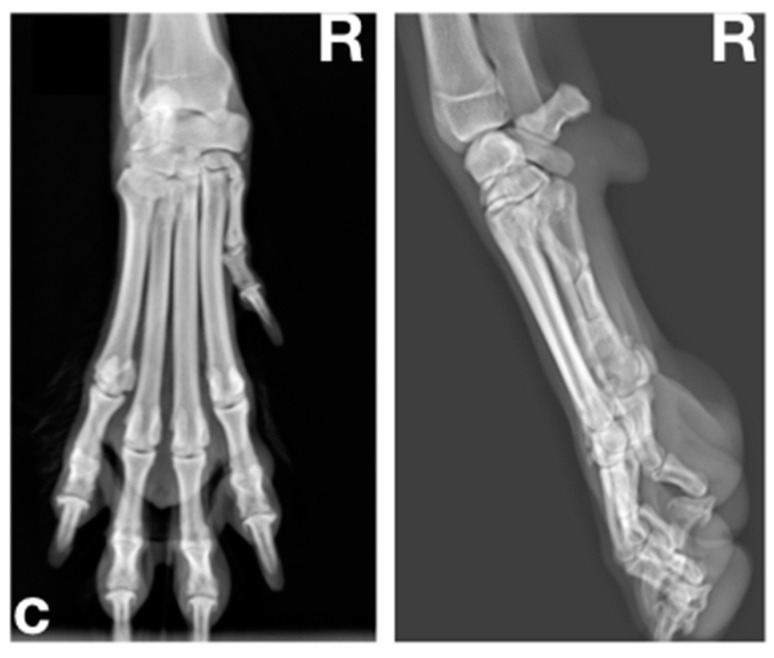
The radiographic assessments of the forepaw of the three patients were within normal limits. (**a**) Case 1 showed no radiographic changes, nor did case 2 (**b**) or case 3 (**c**).

Figure 2 shows the radiographic assessments of the forepaws of the three patients.

**Figure 3:**
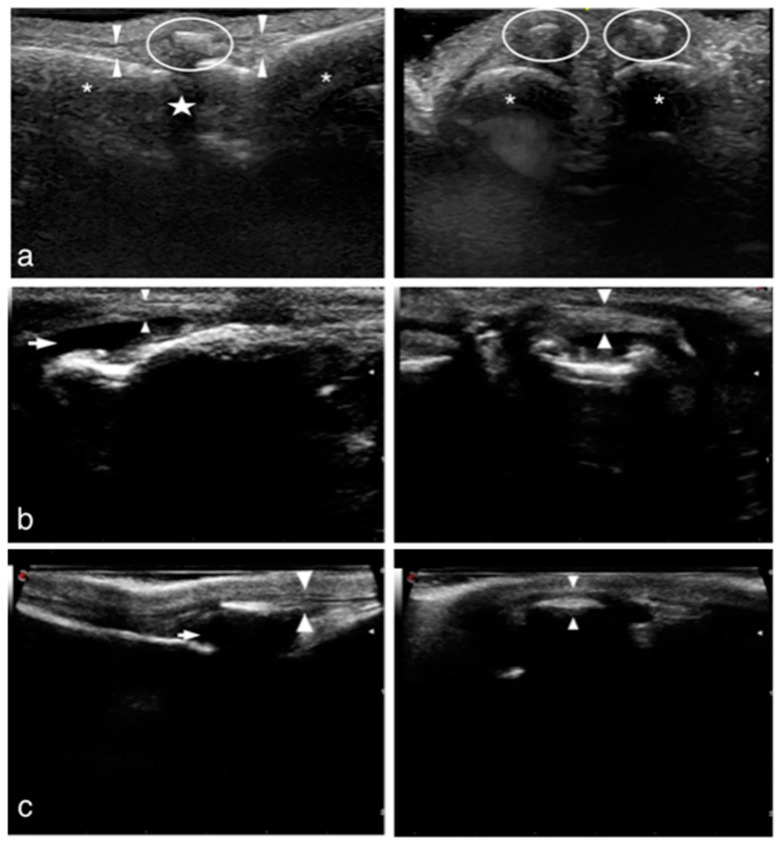

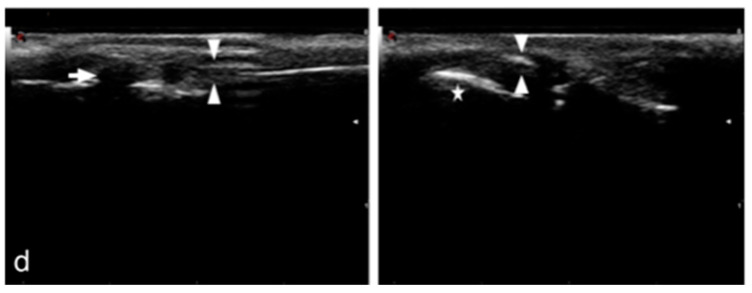
US assessment of the forepaws of the three patients compared with the normal US appearance. (**a**) Normal US anatomy, longitudinal scan: the CDET with its straight and well-defined fibrillar pattern is clearly visualized (between white arrowheads). The embedded sesamoid bone (white circle) is visible. No signs of effusion are present around the tendon. The MCP joint space (white star), the metacarpal bone (right white asterisk), and the first phalangeal bone (left white asterisk) are clearly observed. Transverse scan: the CDET with the embedded sesamoid bones (white circles) is clear and well visualized. The two white asterisks indicate the underlying bone surfaces of the first phalanx. (**b**) Case 1: longitudinal scan. Anechoic layer (white arrow) surrounding the CDET of digit IV; the CDET (between white arrowheads) appears grossly intact, although the fibrillar pattern is not well defined. Transverse scan: the CDET (between white arrowhead) of digit IV appears grossly intact without signs of displacement. (**c**) Case 2: longitudinal scan of digit IV. Anechoic layer (white arrow) surrounding the CDET of the digit; the CDET (between white arrowheads) of digit IV appears grossly intact. The sesamoid bone embedded in the tendon is clearly visualized. Transverse scan: the CDET (between white arrowheads) of digit IV appears grossly intact with the embedded sesamoid bone visible and with no signs of displacement. (**d**) Case 3: longitudinal scan. Anechoic layer (white arrow) surrounding the CDET of digit IV; the CDET (between white arrowheads) appears grossly intact. Transverse scan: the CDET (between white arrowheads) of digit IV (white star) appears displaced from its physiological position. Medial subluxation was suspected.

Figure 3 shows the US examinations of the forepaws of the three patients compared with the normal ultrasonographic appearance.

## 4. Discussion

In the present case report, three cases of lesions to the extensor compartment of the paw, at the level of MCP joint, are described. The lesions affected three sporting dogs and occurred during the sport activity. The clinical presentation and the diagnostic findings of the injury seem to share many characteristics with the spectrum of lesions of the digital extensor tendon described in human medicine and called boxer’s knuckle.

The veterinary literature about the damages of the surrounding tissues of the CDET in dogs is scarce, with only a few references regarding horses in which injuries to several structures of the dorsal aspect of the distal forelimb are described, possibly because of their superficial location. The vulnerable structures include the CDET and lesions reported comprise infections and inflammations mainly caused by direct blunt trauma [10]. In sporting dogs, tendon structures of the paws are reported as injury-prone, especially regarding the flexor compartment. Indeed, the superficial and deep digital flexor tendons of dogs competing in different type of sports seem to be at major risk of injury. Lacerations and elongations to digital flexor tendons lead to different degrees of functional impairment borne by phalanx and the clinical implications of such lesions in sporting patients are well recognized [9,11,12].

To date, no reports of damages to the surrounding structures of the extensor tendons of the forepaw in canine athletes have been published and this is, to the best of the authors’ knowledge, the first report of sport-related damages to the CDET in dogs.

The CDET of dogs crosses the extensor surface of the carpal joints dividing the few tendons that pass over the dorsal surface of the corresponding metacarpal bones, reaching the distal phalanxes of digits II to V, inclusively. Dorsally to the MCP joint, a sesamoid bone, not always present, can be embedded in the tendinous structure. Each tendon, at the end of the proximal phalanx, is reached by a thin extension of the tendon of insertion of the interosseous muscles that cross obliquely from the palmar surface [1]. This small and indistinct anatomical structure is reported to have a stabilizing function on the digital extensor tendon of the corresponding digit [13]. Interestingly, its anatomical course seems to be similar to that of the human lateral band, both arising from the palmar side and reaching the CDET on the dorsal aspect of the digit. In the case of dogs, the structure is slightly proximal on the first phalanx compared with the human one. Anatomical differences between the two species are obvious, with the sesamoid bone not present in humans and the sagittal band, a structure that runs circumferentially around the MCP joint, not well distinguishable in dogs. However, in both humans and dogs, the extensor tendons do not insert directly onto the MCP joint or onto the proximal phalanx. Moreover, they extend the MCP joint through a pulley effect that, in humans, is mainly a prerogative of the sagittal band. This latter is attended in its function by the strong connection with lumbricalis and interosseous tendons that are also of significant importance in the anatomical context of the digits of dogs [4,14].

**Figure 4:**
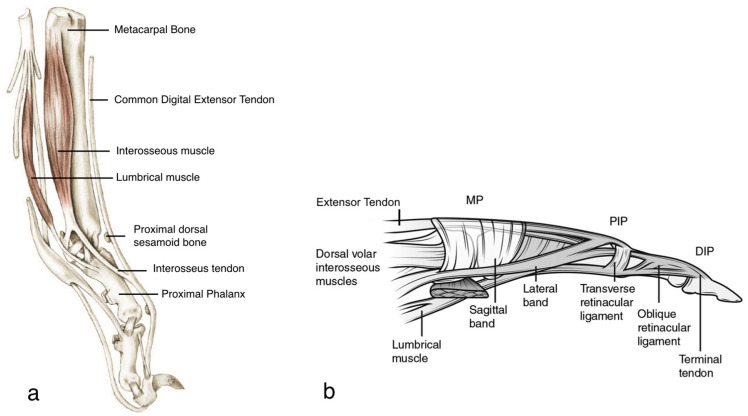
Anatomical comparison between the digits of dog and humans. (**a**) Anatomy of the dogs, medial view. The extensor mechanism at the MCP joint with the interosseus tendon gliding on the dorso-medial aspect of the first phalanx is represented. (**b**) Extensor mechanism at the MCP joint in humans, radial view. The sagittal band arises from the volar plate and intermetacarpal ligament and stabilizes the extensor tendon. DIP: distal interphalangeal joint; MP: metacarpophalangeal joint; PIP: proximal interphalangeal joint. Note the correspondence between the interosseus tendon of dogs and the lateral band of humans.

Figure 4a,b shows the comparison between the anatomy of dogs and humans.

Boxer’s knuckle was first described in human patients in 1957 by Gladden, indicating a spectrum of soft tissue injuries to the dorsal aspect of the hand, secondary to single or repetitive trauma to the metacarpophalangeal joint and the extensor mechanism of the hand and mainly involving the sagittal band [15]. Three degrees of sagittal band tears were described by Rayan et al. in 1994. Type I is described as a simple contusion without tears in the retinacular structures. In this case, tendon instability is not present, and no evidence of luxation or subluxation is described. Type II is associated with extensor tendon subluxation and type III is characterized by clear tendon dislocation. Type III is also associated with an occasional inability to actively extend the involved joint [16].

In human studies, the mechanism of injury is reported to be mainly related to direct blunt traumas, but the concurrence of forced flexion and resisted extension seems to have a predisposing role in the occurrence of the lesion. Thus, sports requiring gripping or grasping, such as tennis, also seem to be more problematic among human athletes [17]. The supposed pathogenesis of the lesion in sporting dogs seems to share many similarities with the injury mechanism in the above-reported human pathology. All three patients of this case series had a history of blunt trauma during sports activity, followed by onset of the clinical signs. Moreover, the mechanism of resisted extension and forced flexion can be a contributing factor in injuries occurred while running. Indeed, all of the dogs were involved in sports with sharp turns and high speed, with a large amount of grip required while running on uneven terrain, all factors that are supposed to be predisposing to distal extremities injuries [18]. Another similarity between the pathology in humans and dogs is the involvement of the central digits in both species. In humans, the middle finger is usually the most involved, followed by the index finger. The knuckle of the middle finger is in fact more prone to injuries because of its position and, interestingly, the affected digit in all three cases of this report was digit IV, with the involvement of digit III in one patient. The localization of the lesion to the central digits of the paw is consistent with previously published studies, which consider digits III and IV of the distal extremities of dogs to be the most overloaded structures during active weightbearing, because of their central anatomical position. The role of digits III and IV of the manus are also well known during the brake phase of sporting activities such as racing, in which the entire body weight of the dog is borne by digits III and IV that are, at the same time, deeply involved in their damper function, while the flexed digits and the extensor compartment are actively involved in the resisted extension reported above [17,19].

The clinical signs observed in the study population of the present case report also seem to share similarities with those described in humans. Dogs showed swelling at the level of the affected MCP joint. Pain at palpation and at full passive forced extension and flexion of the affected digit was also detected. Clear instability of the tendon was present in case 3, whereas case 1 and case 2 exhibited no clear luxation. In people, the clinical presentation is reported to include pain, swelling, and tenderness of the involved anatomical site. In some cases, tendon instability is also present. Active movements of the MCP joints show limitations. Pain increases with full active and passive flexion of the joint. Based on the observed clinical findings, the clinical presentation in dogs would likely seem to be correspondent to that observed in humans.

Diagnoses in human patients are performed with clinical examination with the recognition of all the clinical signs reported above. Radiographic assessments usually show no relevant radiographic signs. The help of other imaging techniques is considered useful, with dynamic high-resolution US of the clenched fist considered to be a reliable tool for the diagnosis of injuries of the extensor hood mechanism [20]. Given the scarcity of data in the veterinary literature and based on those findings extrapolated from the human studies, a similar diagnostic approach was applied in the three cases of the present series. Interestingly, radiographic examinations were also within normal limits in dogs and the US findings showed analogies with that reported in humans. In the present population, US examination of the involved digits showed swelling of the soft tissues, with normal echogenicity of the common extensor tendon. Disruption of the soft tissues surrounding the digital extensor tendon of the involved digit, with irregular echogenicity, was also observed. At the dynamic evaluation, with the digit in complete flexion, a clear displacement of the tendon was seen in case 3, although cases 1 and 2 showed no tendon displacement. In humans, the US evaluation shows grossly intact extensor tendon surrounded by soft-tissue edema. In this species, the dimension of the anatomical structures allows the ultrasonographic visualization of discontinuity, asymmetric thickening, and hypoechogenicity of the involved part of the sagittal band. The clear US observation of the stabilizing structures is not possible in dogs because of their small dimensions. However, the indirect signs of soft tissue disruption are clearly detectable with high-resolution US and, interestingly, might be considered similar to those described in humans [21,22].

**Figure 5:**
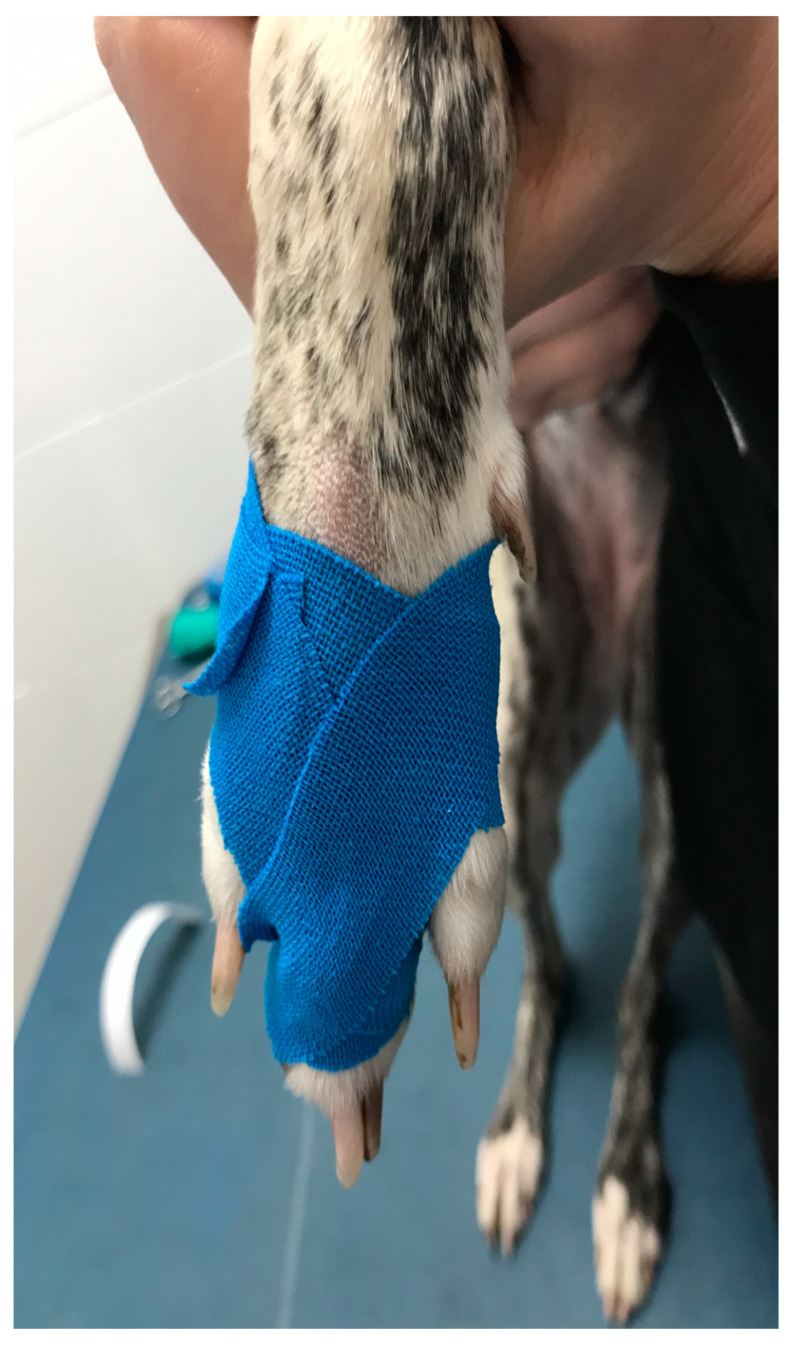
Case 1. Application of the buddy taping technique to patient number 1. The tape is applied fixing the involved digit to the adjacent one in order to take advantage of the splinting action offered by the adjacent intact digit. ROM is only partially limited.

Treatment options in dogs are not reported in the literature. In human athletes, nonsurgical management has been reported from Kleinhenz et al. for the treatment of most type I and type II injuries, which are those without clear tendon luxation, as well as most of the type III injuries. In those cases, a buddy taping technique is applied to the involved finger for four to six weeks. In humans, surgical management is reserved to cases that do not respond to conservative treatment and to cases with chronic clear tendon luxation [23]. Based on those data, a conservative approach with four weeks of buddy taping technique application was the choice. Although reported for treatment of other digit injuries in sporting patients, the technique was applied with the involved finger in extension and fixed to the adjacent one, in order to promote MCP joint stability with the reduction in the articular range of motion that is only partially limited [9,17]. Figure 5 shows the aspect of the taping for case 1.

## 5. Conclusions

Injuries to the extensor compartment of the MCP joint in sporting dogs are not reported in the literature; this paper presents the first description. The anatomy of the involved structures shows similarities between dogs and humans, although considering specific obvious differences. The clinical signs and the US findings observed in the patients described in this case report also suggest an analogy with the pathology reported in human patients and called boxer’s knuckle. As in humans, in dogs too, the pathogenesis of the lesion seems to be mainly related to the occurrence of blunt force traumas during the sporting activity. Biomechanical factors related to the load on the central digit of the manus are supposed to have a role in the occurrence of the lesion. The treatment options are not described in literature and, for the three described cases, a conservative approach already described for digital tendons injuries in dogs with a “buddy taping” technique was chosen, with a satisfactory outcome at three-month follow-up [9].

In conclusion, this small case series suggests to consider lesions to the CDET at the level of MCP joints among the differential diagnosis of thoracic limb lameness in canine sporting patients involved in sports that are prone to blunt trauma. Although no conclusions about treatment can be withdrawn from this small case series and considering that further studies are necessary to better understand the involvement of the extensor compartment of the forepaw in sport-related injuries in canine athletes, this first description of CDET lesion and its treatment in sporting patients gives a new insight to the growing body of sporting dog literature.

## Figures and Tables

**Table 1:** Lameness evaluation system.

Grade	Degree of Lameness
1	No lameness
2	Intermittent weight-bearing lameness
3	Permanent weight-bearing lameness
4	Non-weight-bearing lameness

## Data Availability

All data contained within this article are available. Interested qualified researchers may request additional information.

## Supplementary Materials

The following supporting information can be downloaded at https://www.mdpi.com/article/10.3390/ani12192619/s1, Video S1: Clear CDET instability is detectable in case 3.
